# Predictive ability of Achilles tendon elastography for frailty in older adults

**DOI:** 10.1007/s41999-024-01023-9

**Published:** 2024-08-01

**Authors:** Eda Ceker, Ayse Fadiloglu, Esra Cataltepe, Halit Nahit Sendur, Seriyye Allahverdiyeva, Hacer Dogan Varan

**Affiliations:** 1 Faculty of Medicine, Department of Geriatric Medicine, Gazi University, 06560 Ankara, Turkey; 2 Faculty of Medicine, Department of Radiology, Gazi University, Ankara, Turkey

**Keywords:** Frailty, Achilles tendon elastography, Older adults

## Abstract

**Purpose:**

The Achilles tendon (AT) is the largest and strongest tendon in the human body, and its elasticity is known to be affected by the aging process. However, the relation between AT stiffness and frailty in older individuals remains uncertain. This study aims to explore the potential of Achilles tendon shear wave elastography (AT-SWE) as a tool for assessing physical frailty in older adults.

**Methods:**

A total of 148 patients aged 65 years and over were included in this cross-sectional study. Patients with heart failure, AT injury, stroke history, active malignancy, and claudication were excluded. All patients underwent a comprehensive geriatric assessment. Physical frailty assessment was performed with the fried frailty phenotype. Achilles tendon elastography was measured by ultrasound.

**Results:**

The mean age of the participants was 73.8 years and 62.2% were female. 30.4% of the participants were defined as frail. Achilles tendon shear wave elastography measurements were statistically lower in the frail group (*p* < 0.05). In the multivariate regression analysis, AT-SWE demonstrated a statistically significant association with frailty independent of confounding factors (OR 0.982, 95% CI 0.965–0.999, *p* value = 0.038). In the ROC curve analysis, the area under the curve for AT-SWE was 0.647 (95% CI, 0.564–0.724, *p* < 0.01) and the optimum cut-off point was 124.1 kilopascals.

**Conclusion:**

These findings highlight the value of AT-SWE as a non-invasive and objective tool for predicting frailty in older adults.

## Key summary points

**Aim:**

To investigate the potential of Achilles tendon shear wave elastography (AT-SWE) for assessing physical frailty in older adults.

**Findings:**

Achilles tendon shear wave elastography measurements were statistically lower in frail patients and showed a significant association with frailty after adjustments of age, gender, and chronic diseases.

**Message:**

Further prospective cohort studies should explore the potential value of using AT SWE to diagnose frailty.

## Introduction

The Achilles tendon (AT) is the largest and strongest tendon in the human body, crucial for maintaining normal gait and providing stability to the ankle and foot during weight-bearing activities. Aging leads to significant changes in the AT, including reduced blood supply, alterations in collagen volume, and decreased levels of glycosaminoglycans, resulting in decreased tensile stress, elastic modulus, and stiffness [1, 2]. Various imaging techniques such as MRI, ultrasound, and elastography, particularly shear wave elastography (SWE), offer non-invasive means to assess the structure and elasticity of the AT [3, 4]. Specifically, SWE provides quantitative data on tissue elasticity and stiffness and specialized forms of SWE, such as shear wave tensiometry, hold promise for evaluating balance and fall risk in older adults [5] [6].

Frailty is a geriatric syndrome that identifies individuals with diminished physiological reserve and resistance against stressors, thereby increasing the risk of adverse health outcomes such as disability, morbidity, and mortality. [7–9]. Physical frailty encompasses various manifestations, including reduced muscle strength, flexibility, coordination, and balance, and decreased levels of physical activity[10]. Various measurement tools have been developed for physical frailty, the most widely used of which is the fried frailty phenotype (FFP) by Fried et al. [7]. Slower gait speed, low physical activity, less strength, and exhaustion, are the core components of physical frailty [11].

Aging-related changes in AT stiffness may lead to decreased resistance during movement, affecting walking performance and balance, crucial for maintaining mobility and physical function [12]. Since elastic tendons transfer muscle forces to skeletal bones, it has been proposed that changes in tendon properties also contribute to the age-related decline in functional performance [13]. Reduced tendon stiffness increases the metabolic cost of walking and decreases walking speed, impacting independence and quality of life in older adults [14]. On the other hand, frailty is commonly associated with sarcopenia [15] and sarcopenia may potentially lead to alterations in biomechanical stress on the AT. Thus, with a two-way relationship, frailty itself can contribute to changes in the AT, and changes in the AT can also contribute to frailty. Although the association between reduced AT stiffness and frailty is not firmly established, it is evident that the outcomes coincide with the physical performance indicators of frailty. Furthermore, age-related changes in tendon structure and properties may contribute to functional decline and frailty in older adults.

The musculoskeletal applications of SWE have been previously reviewed and examined, highlighting its potential utility in monitoring age-related changes such as sarcopenia and frailty syndrome[16]. However, to our knowledge, there is currently no research specifically evaluating AT SWE for frailty. Based on the demonstrated impact of decreasing AT stiffness on mobility and physical performance, which are also linked to frailty, we hypothesize that AT stiffness may contribute to functional decline and frailty in older adults.

## Materials and methods

The cross-sectional study protocol was prepared according to the principles of the Declaration of Helsinki. The Local Ethics Committee approved the study protocol and written informed consent was obtained from each participant.

### Study population

This study was conducted at the Geriatrics and Radiology departments of Gazi University, between June 2022 and June 2023. All participants were selected from the Geriatrics outpatient clinic.

A total of 148 patients (men, women, mean age years) were included in the study and scheduled to undergo AT SWE after routine laboratory tests and geriatric assessment were performed. Patients with histories of AT injury, musculoskeletal disease, immobility, rheumatologic disorders, joint prosthesis, peripheral vascular and cerebrovascular disease, familial hypercholesterolemia, active malignancy, and infections were excluded.

All patients underwent a physical examination and a careful evaluation of medical history, comorbidities, and current pharmacologic treatments. Patients’ age, sex, comorbid diseases, drugs used, smoking status, exercise status, body weight, height and body mass index (BMI) were recorded. Complete blood counts, renal and liver functions, cholesterol, albumin, thyroid stimulating hormone (TSH), and vitamin D levels were recorded from routinely collected blood samples.

Anthropometric and physical performance measurements were conducted including height, weight, arm circumference, and calf circumference. Calf circumference was measured at the widest area of the calf, and arm circumference was measured at the mid-arm, using a nonelastic measuring tape. The Takei grip strength dynamometer was used to assess the participants’ muscle strength. Three measurements of grip strength for the dominant hand were taken and the highest measurement was recorded for analysis. The European Working Group on Sarcopenia in Older People (EWGSOP-2) consensus criteria were used to determine low muscle strength, defined as less than 16 kg for women and 27 kg for men [17].

Participants’ physical performance was evaluated using the 6-m walk test. In this test, participants walked at their normal pace and the time taken to complete the distance was measured with a stopwatch. This recorded time was then used to calculate walking speed, expressed in meters per second (m/s).

Frailty was diagnosed using the freid frailty phenotype (FFP)[18]. The FFP includes five domains including unintentional weight loss, exhaustion, weakness (decreased hand grip strength), slow gait speed, and low physical activity. Each criterion is assigned a score of 0–1, with a maximum total score of 5. Individuals with a score of ≥ 3 are classified as frail, a score of 1 or 2 indicates prefrailty, and finally, a total score of 0 is considered robust, indicating a higher level of physical resilience.

### Achilles tendon ultrasonography

Achilles tendon examinations were conducted by two experienced radiologists using a high-resolution ultrasound system (RS85, Samsung Medison Co. Ltd.) equipped with a linear transducer (2–9 MHz). The evaluation was performed in B mode while the patient lay in the prone position with both feet hanging over the examination bed in a relaxed position, with the ankle at approximately 90° plantar flexion. The Achilles tendon was examined longitudinally by placing the linear probe approximately 2 cm proximal to the calcaneus insertion (middle region of AT, 2–6 cm above the insertion on the calcaneus), without applying pressure.

**Fig. 1:**
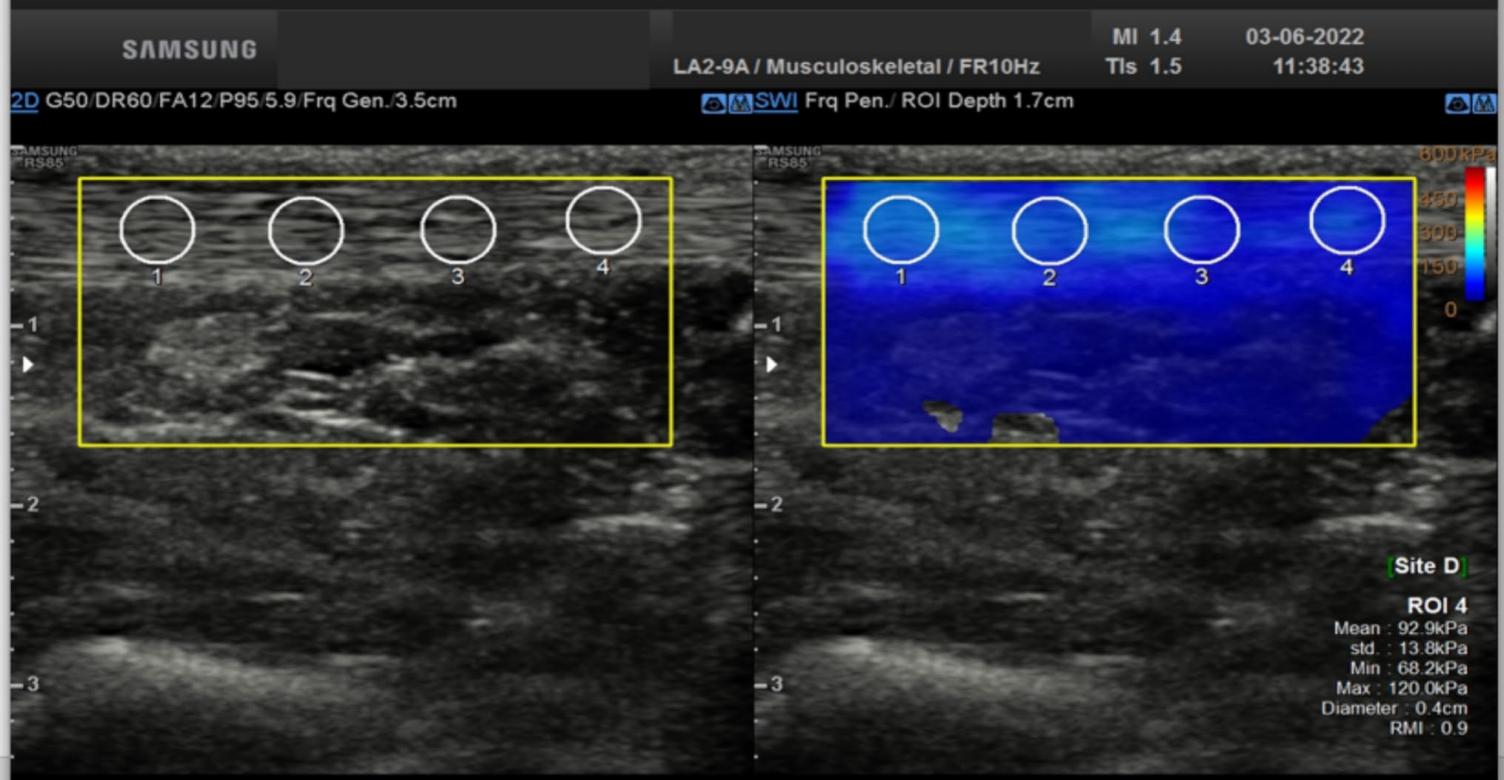
The Achilles tendon shear wave elastography measurement with four ROI and color grades

Achilles tendon thickness (AT-T) was determined by measuring the maximum distance between the anterior and posterior walls. AT-SWE evaluations were conducted in the same position, covering the entire AT thickness. Three measurements were taken for each patient, with four regions of interest (ROI) in each phase (Fig. 1). The diameters of ROIs were approximately 4 mm and the mean value of these four ROIs was recorded in kilopascals (kPa). Finally, the mean value of the three consecutive mean stiffness measurements was used for the analyses.

### Statistical analysis

All analyses were performed using SPSS 22.0 (SPSS for Windows 17.0, Chicago, IL, USA). The data were expressed as the means ± the SDs for continuous variables and as percentages for categorical variables. The Kolmogorov–Smirnov test was used to determine whether the continuous variables were normally distributed. Normally distributed continuous variables were compared using the Student’s *t* test. Categorical variables were compared using the Chi-square test. Spearman’s correlation was used to examine the relationships between the AT SWE measurements and the continuous variables. Multivariate logistic regression analysis was used to determine the independent predictors of frailty. Significant parameters in the univariate analysis were included in the multivariate model.

A receiver operator characteristic (ROC) curve analysis was performed using MedCalc v22.009 to identify the optimal cutoff points of AT SWE for frailty. The area under the curve was calculated to test the accuracy of the analysis. A *p* value < 0.05 was considered statistically significant.

## Results

The study included a total of 148 patients, with 45 classified as frail (30.4%) according to FFP. Among the participants 56 (37.8%) were male. Statistically significant differences were not found between genders in terms of both frailty rates (*p* = 0.064) and median AT SWE measurements (*p* = 0.145). The median value for women was 127.35 kPa (min–max: 80.2–212.7) and 138.5 kPa for men (min–max: 67.9–273.0). In terms of comorbidities, 70.5% of the patients had hypertension (HT), 43 (29.1) % had diabetes mellitus (DM), 39 (26.4%) had coronary artery disease (CVD). Table 1 presents the general characteristics of all patients, as well as those grouped according to frailty status based on FFP.

**Table 1:** The characteristics of patients and AT thickness and AT-SWE values

	Total *n* = 148	Non-frail *n* = 103	Frail *n* = 45	*p* value
Gender				
Female, *n* (%)	92 (62.2)	59 (57.3)	33 (73.3)	0.064
Male, *n* (%)	56 (37.8)	44 (42.7)	12 (26.7)	
Age, mean (SD)	74.31 (6.56)	72.83 (6.51)	77.68 (5.38)	< 0.01
Smoking status, *n*, (%)	53 (35.9)	40 (38.8)	13 (28.9)	0.246
Falls in last year, *n*, (%)	42 (28.4)	25 (24.3)	17 (37.8)	0.094
Exercise status				
Non	60 (40.5)	33(32)	27 (60)	0.005
1–2 day/week	31 (20.9)	23 (22.3)	8 (17.8)	
3 or more day/week	57 (28.5)	47 (45.6)	10 (22.2)	
HT, *n*, (%)	105 (70.9)	68 (66)	37 (82.2)	0.046
DM, *n*, (%)	43 (29.1)	24 (23.3)	19 (42.2)	0.02
HL, *n*, (%)	41 (27.7)	27 (26.2)	14 (31.1)	0.540
CVD, *n*, (%)	39 (26.4)	27 (26.2)	12 (26.7)	0.954
Number of medicines used, median (min–max, IQR)	3 (0–11, 4)	3 (0–9, 4)	3 (0–11, 3)	0.02
Height, cm, mean (SD)	156.61 (9.66)	152.27 (9.61)	157.88 (9.35)	< 0.01
Weight, kg, mean (SD)	68.8 (13.22)	71.9 (12.45)	61.85 (12.3)	< 0.001
BMI, kg/m^2^, mean (SD)	28.11 (5.14)	28.8 (4.92)	26.45 (5.30)	0.009
Arm circumference, mean (SD)	28.79 (3.52)	29.49 (3.38)	27.2 (3.34)	< 0.001
Calf circumference, mean (SD)	35.96 (4.35)	36.72 (3.86)	34.22 (4.92)	0.001
Handgrip strength, median (min–max, IQR)	19.25 (6.8–41.3, 10.6)	20.9 (6.8–41.3, 10.05)	15.6 (11.7–28.8, 4.5)	< 0.001
Gait Speed, m/s, median (min–max, IQR)	1.03(0.38–2.82, 0.46)	1.15 (0.57–2.82, 0.36)	0.66 (0.38–1.24, 0.4)	< 0.001
AT thickness, mm, median (min–max, IQR)	5.09 (3.16–7.6, 1.36)	5.08 (3.16–7.6, 1.47)	5.14 (3.73–7.1, 1.01)	0.111
AT-SWE, kPa, median (min–max, IQR)	129.8 (67.9–247.4, 56.1)	131.3 (67.9–247.4, 56.2)	114.6 (74.2–203.1, 40.7)	0.003
ADL, median (min–max, IQR)	6 (5–6, 1)	6 (5–6, 1)	6 (5–6, 1)	0.077
IADL, median (min–max, IQR)	8 (3–8,1)	8 (5–8, 1)	7(3–8, 3)	< 0.001
MNA, median (min–max, IQR)	12 (3–14,4)	13 (7–14, 2.5)	10 (3–14, 3)	< 0.001
MMSE, median (min–max, IQR)	28 (16–30,3)	29 (21–30, 3)	26(18–30, 6)	< 0.001
GDS, median (min–max, IQR)	2 (0–13, 4)	2 (0–9, 4)	2 (0–13, 7)	< 0.001
TSH, µIU/ml, median (min–max, IQR)	1.61 (0.01–8, 1.27)	1.74 (0.01–8, 1.42)	1.41 (0.11–7.91, 1.34)	0.166
Uric acid, mg/dl, mean (SD)	5.43 (1.41)	5.43 (1.35)	5.43 (1.55)	0.99
CRP, mg/L, median (min–max, IQR)	3.51 (1–93.8, 4.3)	3.43 (1–44.4, 7.94)	3.57 (1–93.8)	0.436
Total Cholesterol, mg/dl, mean(SD)	205.12 (46.44)	208.11 (49.1)	198.28 (39.16)	0.23
Albumin, gr/dl, median (min–max, IQR)	4.3 (3–6.5, 0.3)	4.2 (3.3–5, 0.3)	4.3 (3–6.5, 0.5)	0.577

*ADL* activities of daily living, *AT* Achilles tendon, *AT-SWE* Achilles tendon shear wave elastography, *BMI* body mass index, *CRP* C-reactive protein, *CVD* chronic cardiovascular disease, *DM* diabetes mellitus, *GDS* geriatric depression scale, *HT* hypertension, *HL* hyperlipidemia*, IADL* ınstrumental activities of daily living, *MMSE* mini-mental state examination,* MNA* Mini nutritional assessment, *TSH* thyroid stimulating hormone

**Fig. 2:**
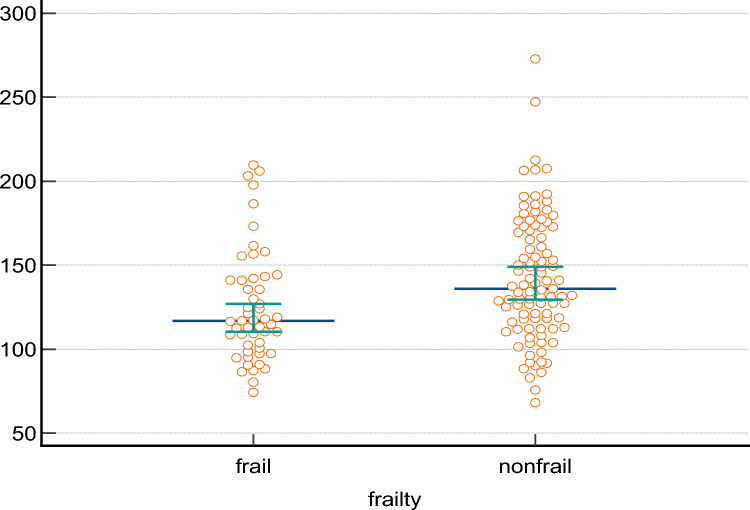
AT-SWE measurements in frail and non-frail groups

Significant differences were observed between frail and non-frail patients in terms of age, weight, BMI, arm circumference, calf circumference, handgrip strength, and gait speed (*p* < 0.01). Achilles tendon SWE measurements were significantly lower in the frail group (*p* < 0.01) (Fig. 2), while there was no difference in AT thickness between frail and non-frail groups.

**Fig. 3:**
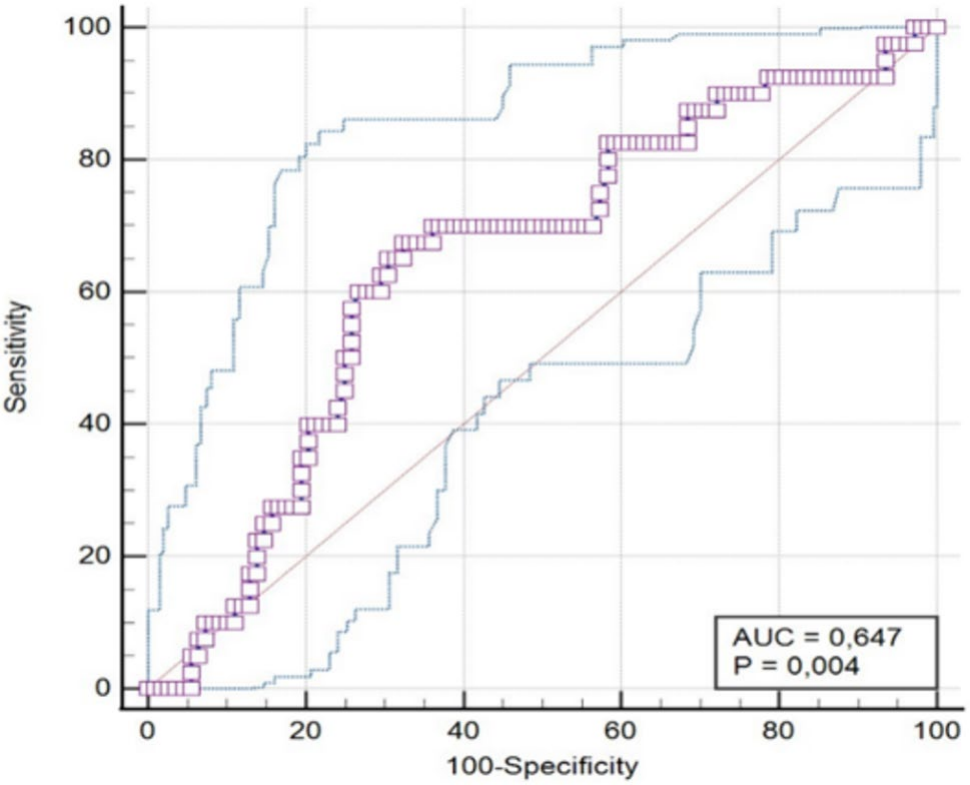
The ROC curve analysis of AT-SWE for frailty in the general population

The diagnostic accuracy of AT-SWE for frailty in the general population was assessed using ROC curve analysis, which determined that the optimal cut-off value for AT-SWE is ≤ 124.1 (Fig. 3). The area under the curve (AUC) was 0.647 (SE 0.05, *p* value: < 0.01, 95% CI 0.564–0.724). Sensitivity was 67.5% and specificity was 67.59% with an 81.4 negative predictive value.

A significant weak negative correlation was found between AT-SWE and age (0.209, 0.011) and FFP score (0.246, *p* = 0.003). Similarly, a weak but significant positive correlation was observed with handgrip strength (0.185, *p* = 0.025), and gait speed (0.230, *p* = 0.005).

Regression analysis was conducted with three models to investigate the relationship between AT-SWE and frailty, as well as the confounding factors for frailty. Model 1 consisted of age and gender. Model 2 included age, gender, DM, CVD, HT, thyroid hormone replacement therapy for hypothyroidism, and the number of medicines used. In model 3, parameters that were found to be significant in the univariate regression analysis or those relevant in the literature with AT-SWE were included. In all models, AT-SWE maintained a significant relationship with frailty. In all models, AT-SWE maintained a significant relationship with frailty. The regression models and analysis results are displayed in Table 2.

**Table 2:** Regression models and analysis for frailty

	Independent variables	OR	95% Cl	*p*
Adjusted model 1	AT-SWE	0.988	0.976–0.999	0.032
	Age	1.127	1058–1201	< 0.001
	Gender	0.505	0.220–1.158	0.107
Adjusted model 2	AT-SWE	.987	.976–.999	.032
	Age	1.119	1.047–1.196	.001
	Gender	1.798	.746–4.334	.191
	DM	1.534	.583–4.035	.386
	CVD	.727	.270–1.958	.528
	HT	1.068	.367–3.109	.903
	Hypothyroidism treatment	.416	.111–1.555	.192
	Number of medicines used	1.176	.922–1.500	.191
Adjusted model 3	AT-SWE	0.982	0.965–0.999	0.038
	Age	1.046	0.959–1.142	0.308
	Exercise status			
	Non	1	1	0.504
	1–2 day/week	2.007	0.580–6.948	0.271
	3 or more day/week	1.894	0.416–6.689	0.359
	DM	0.629	0.186–2.127	0.456
	HT	1.670	0.433–6.434	0.456
	BMI	1.085	0.908–1.298	0.368
	MNA Score	0.628	0.495–0.797	< 0.001
	IADL	0.514	0.320–0.825	0.006
	Arm circumference	0.833	0.638–1.088	0.180
	Calf circumference	1.035	0.865–1.240	0.705
	Number of medicines used	1.203	0.910–1.589	0.194

*AT-SWE* Achilles tendon shear wave elastography, BMI body mass ındex, *CVD* chronic cardiovascular disease,* DM* diabetes mellitus, *HT* hypertension,* MNA* mini-nutritional assesment

## Discussion

To our knowledge, this is the first study to investigate the relationship between Achilles tendon elastography and frailty. We observed a significant difference in AT-SWE between frail and non-frail individuals. Although AT-SWE measurements was found to be significantly lower in the frail group and there was a significant negative correlation with FFP score, we could not find a significant relationship between AT thickness and frailty, suggesting that SWE may provide unique information beyond structural changes. Moreover, regression analysis demonstrated that AT-SWE maintained a significant relationship with frailty even after adjusting for age, gender, and chronic diseases or the number of medicines used.

Shear wave elastography, showed a high level of agreement between operators, making it a reproducible technique for quantitatively assessing the mechanical properties of the AT. Using a longitudinal plane and a relaxed foot position, measures were shown to be even more reliable [19]. In this context, we also conducted measurements, using a longitudinal plane and a relaxed position.

The body’s largest and strongest tendon AT, connects the calf muscles to the heel bone [4], and is susceptible to degenerative changes and altered stiffness due to its frequent use and strain during physical activities. Age and gender-related changes in AT stiffness have been previously examined, revealing a decrease in stiffness with age [13, 20–22]. Consistent with previous findings, we also found a negative correlation between age and AT SWE measurements.

Previous studies conducted with young adults examining the relationship between gender and AT flexibility have yielded conflicting results. In some studies, it is claimed that men tend to have stiffer tendons compared to women in young [23] and older adults [24], and this difference attributed to changes in estrogen levels and force production abilities [23]. However, another study also found differences in the AT in terms of stiffness, tendon elongation, and strain between adult men and women, but statistical analysis revealed that the difference, was correlated to the difference in muscle strength rather than gender [25]. We did not observe a statistically significant difference in tendon stiffness between genders. This discrepancy may be due to differences in the studied populations. To address the confounding effect of the increasing incidence of frailty with age [11] and gender-related tendon stiffness in older adults previously reported in the literature, we performed regression analyzes adjusting for age and gender. It was observed that the significant relationship between frailty and AT-SWE persisted.

Tendon stiffness, which describes the relationship between applied force and tendon’s length change, significantly influences tendon kinematics during movement and enhances motion [25]. Age-related changes in AT stiffness have been shown to affect walking speed in older adults, which is a parameter of FFP [14]. We observed a weak but significant positive correlation with gait speed (0.230, *p* = 0.005), indicating that walking speed increases as the AT stiffness increases.

Although several studies have implicated the change of AT stiffness in various populations, including individuals with gout [26], hypothyroidism [27], DM [28], and cardiovascular disease [29], the significance of AT-SWE persists even after adjusting for CVD, DM, HT, hypothyroidism treatment, and the number of medicines used in different models of regression analyses. These results therefore strengthen our hypothesis by showing that the association between AT and frailty is independent of common parameters shared with low AT stiffness, such as gait speed decline, and the multimorbidity associated with frailty.

Our study has several strengths. Primarily, it is the first study in the literature to examine the relationship between AT and frailty. Secondly, we try to carefully exclude the factors espically diseases with physical consequences that could influence the investigation of the relationship between AT and frailty. However, the study has some limitations. One of these is the cross-sectional design of the study. Longitudinal, prospective studies are the ideal approach, but they would be impractical for understanding the effects of aging and other confounding factors on AT stiffness. In this context, we were unable to assess changes in patients’ weight, the severity and chronic course of diseases until their current age, exercise habits throughout their lives, and variations in biomechanical load on the AT due to working conditions. Additionally, medications such as corticosteroids, quinolone antibiotics, aromatase inhibitors, and HMG-CoA reductase inhibitors used in comorbid diseases throughout life can affect the AT, often causing tendinopathy [30] and the other muscles and tendons may also be affected by one or a combination of these drug classes. Although the current results include chronic diseases and laboratory values that could affect tendon structure, we could not analyze recent and past drug use histories. Given the challenges of long-term monitoring of all these factors, cross-sectional designs with larger sample sizes covering a wide age range would be valuable.

In conclusion, our findings suggest that reduced AT stiffness is associated with frailty and may serve as a valuable marker for identifying individuals at risk of adverse health outcomes. Further research is needed to elucidate the underlying mechanisms and clinical implications of this relationship. AT SWE may offer a novel perspective on frailty and develop interventions to prevent or mitigate frailty-related functional decline in older adults.

## Data Availability

All data generated or analyzed during this study is available for requests and can be obtained from the corresponding author.
